# Higher rates of food insecurity and stress experienced by food systems workers during the first year of the COVID-19 pandemic

**DOI:** 10.3389/fnut.2024.1274656

**Published:** 2024-05-07

**Authors:** Emma H. Spence, Meredith T. Niles, Farryl Bertmann, Teresa Mares, Emily H. Belarmino

**Affiliations:** ^1^Food Systems Program, University of Vermont, Burlington, VT, United States; ^2^Department of Nutrition and Food Sciences, University of Vermont, Burlington, VT, United States; ^3^Gund Institute for Environment, University of Vermont, Burlington, VT, United States; ^4^Department of Anthropology, University of Vermont, Burlington, VT, United States

**Keywords:** food system, worker, stress, mental health, food security, COVID-19 pandemic

## Abstract

**Introduction:**

The present study examined the prevalence of food insecurity and perceived stress among food system workers relative to other members of the population during the first year of the COVID-19 pandemic. It also explored perspectives on the role of food system workers during the pandemic and their experiences working during this time.

**Methods:**

Data were collected via an online survey in spring 2021. The sample was comprised of 441 residents of Vermont, United States, including 41 food system workers.

**Results:**

Regression models identified higher rates of food insecurity and perceived stress among food system workers during the first year of the pandemic. However, these relationships were not maintained when the models were adjusted for income and job disruption, suggesting that the associations were primarily due to the economic vulnerability of food system workers. Most respondents indicated concern for the health and well-being of food system workers, felt that food system workers were undervalued, and agreed that the well-being of food system workers should be prioritized. However, opinions were split regarding whether it was worth the health risk to require farms and food processing plants to stay open to maintain the food supply. Half of food system workers believed that their work had compromised their well-being during the pandemic, although several also identified their jobs as pathways for accessing food.

**Discussion:**

The findings provide valuable information for decision-makers seeking to increase the resilience of the food supply and the food system workforce.

## 1 Introduction

The 2019 novel coronavirus disease (COVID-19) pandemic and the resulting economic impacts exposed vulnerabilities in supply chains and caused significant hardship. Across the world, the importance of food and agricultural workers to ensuring the stability of the food supply was thrust into the public eye (1, 2). Food system workers, including grocery store clerks, farmers and farmworkers, and more, risked their health to cultivate, process, stock, and deliver food to our tables thus becoming synonymous with other groups of “essential” workers during the pandemic (3, 4).

Around the world, policy exemptions were implemented to allow food system workers to continue to do their jobs (5, 6). In the United States (U.S.), the federal government temporarily expanded the H-2A visa program, which grants migrant farmworkers temporary visas to enter the country, in an effort to ensure continued food production (7). Even as states implemented stay-at-home orders, agricultural workers were instructed to carry on for the public good (8–10). At the same time, other food workers found their hours and income reduced and struggled to make ends meet (11). Alongside a significant increase in demand for food retail options, the U.S. food service industry experienced a notable drop in demand (3). Some farmers, unable to adapt to rapidly changing demand parameters, were forced to dispose of entire fields of crops (3) or livestock products (12). While many of these immediate impacts have evened out over time, the challenges faced by food system workers during this time laid bare preexisting inequalities in the US (13) and beyond (6).

To date, few studies have examined the impact of the pandemic on the food security and well-being of food system workers during the pandemic in-depth. The aims of this study are (1) to analyze rates of food insecurity and stress among food system workers in Vermont, United States a year after the start of the pandemic and (2) to understand how food system workers perceive and characterize their own experiences during the pandemic.

### 1.1 United States food system workers during the COVID-19 pandemic

Food and agricultural workers have been hailed as heroes in the popular media, yet efforts to ensure their protection during the pandemic were limited (7, 13). Agricultural and food processing workers have experienced COVID-19 outbreaks at rates significantly higher than the general public (7). As early as April 2020, roughly 12,000 COVID-19 cases and 48 deaths had been reported among US meat and poultry processing workers alone (3). Elevated infection rates relative to the surrounding community have also been reported in grocery workers (14). For example, in a May 2020 study of one grocery store in Massachusetts, 20% of workers (*n* = 104) tested positive for COVID-19; notably, workers in positions with direct customer interaction were 5 times more likely to test positive (15). Close proximity to others is a common requirement in many food systems jobs, which creates additional safety challenges (14).

After some early outbreaks, state and federal governments made efforts to enhance protections for food system workers. Multiple federal establishments collaborated to issue COVID-19 Guidance for Agricultural Workers and Employers, but the majority of implementation was left to state and local institutions (7). For example, in Oregon, the state Occupational Safety and Health Administration (OSHA) implemented temporary regulations in an attempt to formalize implementation of 6-ft social distancing guidelines in the food and agricultural sector (16). However, such regulatory attempts failed to account for the unique challenges associated with much work in the industry. Maintaining 6-ft distances between workers in farming and food processing industries can be all but impossible, particularly in cases where farmworkers share housing (8, 10, 17). Even where possible, 6-ft distances may be insufficient to protect workers under conditions of poor ventilation and extended exposure (16). Provision of personal protective equipment (PPE) was late and insufficient for many in agriculture, food processing and food retail industries (8, 18, 19). Additionally, testing and screening procedures have been limited in farm and food processing industries (13, 16). Health and safety measures have direct consequences for workers. For instance, in a series of surveys of safety practices in 319 grocery stores, Crowell et al. (18) found that stores with higher enforcement of mask usage, restrictions on reusable bags, and responsiveness to worker complaints showed lower COVID-19 infection rates.

Many food and agricultural workers lack adequate agency in their professional capacity to ensure safe working conditions (10, 14, 20). A high prevalence of low-wages, part-time work, low job security and limited benefits throughout much of the food sector contributes to imbalanced power dynamics between workers and employers, limiting the ability to negotiate paid sick leave and other protective measures (10, 14). According to 2017 data, roughly 17% of frontline food service workers reported household incomes below the federal poverty level (FPL), and another 39% reported incomes between 100 and 250% of FPL (20). Access to healthcare services and insurance have also historically proved challenging for agricultural and food service workers (20, 21). A significant body of early research has focused on migrant and seasonal farmworkers (MSFWs) during COVID-19 as highly vulnerable food system workers (7, 22). However, beyond the justified interest in migrant and seasonal workers during COVID-19, little published research to date has focused on the experiences of food workers. This research aims to expand these analyses by incorporating the experiences of other food system workers, primarily those in the food retail and food service sectors.

### 1.2 Hunger and food insecurity among food system workers in the United States

The United Nation’s Food and Agriculture Organization (FAO) defines food security as a multidimensional concept incorporating food availability, accessibility, utilization, and stability over time (23). The U.S. Department of Agriculture (USDA) describes food security as “consistent, dependable access to enough food for active, healthy living” (24). While numerous population-level studies in the U.S. found increased rates of food insecurity during the COVID-19 pandemic (25–27), assessments of food security among food system workers are rare. However, several studies have examined characteristics of essential workers, a category which includes many food system workers, during the COVID-19 pandemic. Clay and Rogus (11) found in their cross-sectional survey of New York state residents (excluding the New York City metro area), that, in a multivariate model, essential worker status was associated with increased challenges in food access. Additionally, essential workers have a lower average education level, earn lower wages and are more likely to be black, Indigenous, or people of color (BIPOC), which correspond with risk factors for food insecurity (4, 10, 11, 14, 28). Within the retail industry, including grocery, approximately 71% of cashiers are female (14). Parks et al. (3) classify food system workers as among the most economically vulnerable populations. This vulnerability is particularly concerning in the context of employment changes associated with the COVID-19 pandemic. While some food system workers continued working, often in high-risk conditions, others faced reduced hours, furlough, and layoffs; Cho et al. (29) report that “the probability of continued active employment for previous workers” was reduced in both food manufacturing and grocery (p. 3). However, employment reductions were especially pronounced in the food service industry. This is a consequence of both facility closures, and responses to elevated infection risks, which varied by locale (29). Given already low wages, reduced work hours and job loss are more likely to have significant adverse impacts on these workers, elevating the risk of food insecurity (20).

### 1.3 Mental health of food system workers in the United States

Mental health encompasses a person’s psychological, emotional, and social well-being (30). Among other food system workers, farmers and farmworkers are known to experience high rates of stress, depression, and suicide (21, 31). These outcomes may have been exacerbated by added stressors during the pandemic. Clay and Rogus (11) report that those at high risk for contracting the virus and those with financial security concerns are particularly likely to experience anxiety and depression during the pandemic. An online questionnaire of essential workers in Spain—including food system workers—(32) found that 65.2% of grocery workers (*n* = 89) showed a severe psychological impact from the pandemic relative to the general population. Fears of infection and of infecting others have been major themes in several studies of grocery workers (32, 33). In a series of interviews with food retail, food service, and hospitality workers (*n* = 27), Rosemberg et al. (33) found heightened levels of mental distress related to these and other worries. Grocery workers unable to practice social distancing at work have reported higher rates of anxiety and depression than their coworkers (15). Bufquin et al. (34) report that working restaurant employees experienced higher rates of psychological distress and substance use than furloughed employees.

For those food system workers who have experienced food insecurity during the pandemic, mental health outcomes are likely more severe. Food insecurity has been associated with numerous negative physical and mental health outcomes (22, 35). In a systematic review, Bruening et al. (36) found that 83% of studies examined reported an association between negative emotional well-being and food insecurity over time, with the sum of evidence suggesting a bi-directional relationship. More specifically, a meta-analysis of data from 19 studies showed a significant relationship between food insecurity and depression risk and stress, although the association did not hold for anxiety (37).

## 2 Methods

### 2.1 Data collection

The National Food Access and COVID Research Team (NFACT) site based at the University of Vermont collected data for this research (26). The group has administered multiple iterations of a common survey, updated for relevance with each cycle. The survey is concerned with food access and food security at various time points during the COVID-19 pandemic, and also collects data on a variety of relevant demographic and lifestyle factors (26). This study is based on data collected through a statewide survey administered in March–April of 2021. This version represents the third in a series of longitudinal surveys administered to the same convenience sample and was tailored to ask specific questions regarding food system work. Only individuals who completed in the first two surveys were invited to complete the third survey. Informed consent was obtained from all study participants.

### 2.2 Relevant variables

Select demographic and lifestyle factors (Table 1) serve as controls for this study. Variables (e.g., gender) are primarily analyzed in binary form due to small sample sizes. In most cases, demographic variables were collected at the first timepoint in the longitudinal series (March–April 2020) and matched to respondents who remained involved at later timepoints. However, some variables (e.g., income) were collected again in the third survey, in which case the most current data available is used. Additionally, a single variable was created to reflect full or part-time employment in the food system at any point since the COVID-19 pandemic. Participants who reported exclusively volunteer work in the food system were excluded given that volunteers’ tasks and projects are typically of more limited scope.

**Table 1:** Complete list of variables, questions and scales used in analysis.

Variable	Survey question	Scale
*Demographic variables*		
Female	Which of the following best describes your gender identity?	1 = Female, 0 = Not Female*
Income	Which of the following best describes your household income range in 2019 before taxes?	0 = Under 50 k 1 = Over 50 k
Children	Are there children in your household?	0 = No children in HH 1 = Yes, children in HH
Job disruptions	Have you or anyone in your household experienced a loss of income or job since the COVID-19 outbreak (March 11^th^, 2020)?	1 = Yes, 0 = No*
*Food security and stress variables*		
Food Security in the last 30 days	Determined based on the responses to the US Household Food Security Survey Module Six-Item Short Form.	1 = Food Insecure, 0 = Food Secure
Food Security since June, 2020	Determined based on the responses to the US Household Food Security Survey Module Six-Item Short Form.	1 = Food Insecure, 0 = Food Secure
Perceived Stress Scale	Perceived Stress Scale Score calculated based on responses to: In the last month, how often have you felt that you were unable to control the important things in your life? In the last month, how often have you felt confident about your ability to handle your personal problems? In the last month, how often have you felt that things were going your way? In the last month, how often have you felt difficulties were piling up so high that you could not overcome them?	0–16 (higher scores reflect higher stress)
*Federal nutrition assistance program participation variables*		
SNAP Participation	Has your household used SNAP benefits in the last 30 days?	1 = Yes, 0 = No
Food Pantry Utilization	Has your household used a food pantry in the last 30 days?	1 = Yes, 0 = No
*Food system work experience variables*		
Perceived Well-Being of Food System Workers	At any point since the beginning of the COVID-19 pandemic, have you ever felt that your work in the food system compromised your well-being?	1 = Yes, 0 = No
Additional Comments		Open-ended
Food Access of Food System Workers	Has your employment in the food system offered additional pathways to access food?	1 = Yes, 0 = No
Additional Comments		Open-ended
Other Reflections by Food System Workers	Is there anything else you would like to share about your experience working in the food system during or just prior to the start of the COVID-19 pandemic?	Open-ended
*Perspectives on food system workers*		
Health Risks	It is worth the health risk to maintain the food supply such as requiring farms and food processing plants to stay open, because we need food	0 = Disagree, 1 = Agree
Worker well-being	I feel that the well-being of food workers should be prioritized despite potential food supply disruptions	0 = Disagree, 1 = Agree
Worker spread	I am concerned that food workers may spread the virus through their work activities.	0 = Disagree, 1 = Agree
Worker health	I am concerned about the health and welfare of food workers	0 = Disagree, 1 = Agree
Undervalued workers	I feel that food workers are undervalued for the services they provide.	0 = Disagree, 1 = Agree

For these variables, the start of the outbreak is defined as March 11, 2020. *Original categories were condensed due to small sample sizes.

These variables were examined in relation to two primary dependent variables based on self-reported data: food security and perceived stress. We use the USDA validated 6-item short-form food security module to assess food security of participants over the 30 days prior to completing the survey, as well as since June 2020 to achieve a more nuanced analysis of food security since the start of the pandemic (38). The instrument focuses on a household’s financial resources of food and is an abbreviated version of the USDA’S 18-item questionnaire, which is considered the gold standard for measuring food insecurity severity (39, 40). Following standard scoring procedures, those who responded positively to two or more questions are classified as food insecure. We measure perceived stress over the 30 days prior to survey completion using the validated, 4-item perceived stress scale (41). The instrument asks participants to indicate the frequency with which they experienced different scenarios on a scale of 0 (never) to 4 (very often): unable to control the important things in their life, confident about their ability to handle their personal problems, things were going their way, and difficulties were piling up so high that they could not overcome them. To calculate a perceived stress score (0–16), we then sum the responses to each question, first inverting relevant responses so that lower scores consistently reflect lower stress and higher scores reflect higher stress.

Participants who self-identified as food system workers were asked several additional binary (yes/no) questions regarding their experience in this capacity during the pandemic and given the opportunity to expand on these responses in open-ended comments. Topics included reflections on the impact of their work in the food system during the pandemic on their overall sense of well-being, any additional pathways to accessing food offered through their employment, and space to reflect on any additional topics related to their work.

All participants were also asked to respond to a series of questions reflecting their perspectives on the role of food system workers during the pandemic, particularly as it corresponded to the safety and security of the food supply. These questions, as answered both by food system workers and others, are examined in addition to optional qualitative follow-up comments.

### 2.3 Data analysis

We use Chi-Square and T-tests to examine the relationships between food system work and select demographic and outcome variables, depending on the distribution of the outcome variables. We use logit and ordinary least square regression models to predict food security status and perceived stress, using food system work as an independent variable, both unadjusted and adjusted for relevant covariates including income and job loss or reduction.

To predict food security in the last 30 days and in the period since June 2020:

Unadjusted: log(π/1 − π) = β0 + β1(type of job).

Adjusted: log(π/1 − π) = β0 + β1(type of job) + β2(income) + β3(job disruption).

To predict perceived stress:

Unadjusted: perceived stress score = β0(type of job).

Adjusted: perceived stress score = β0(type of job) + β1(income) + β2(job disruption).

For all tests, significance is evaluated at an alpha level of 0.05. Logit regression models predicting food security are reported in odds ratios. Open-ended survey responses were thematically analyzed into *a priori* codes based on the subject matter of the corresponding question and further coded into emergent sub categorical themes where relevant.

## 3 Results

A total of 441 Vermont residents (‘Vermonters’) responded to the March–April 2021 survey and thus were included in analyses. Forty-one of respondents (9.3%) self-identified as food system workers. Food system workers represented the following industry sectors, with several respondents engaged in more than one sector (Supplementary Table 1): food service (16); food retail (10); food processing (8); agriculture (6); and other (3). Among all respondents, similar rates of food insecurity were documented in the past 30 days (18.5%) and in the full period since June 2020 (20.8%). On a scale of 0–16, where a higher value represents greater perceived stress, the average perceived stress score was 5.7 (SD = 3.2), Table 2 presents select demographic and lifestyle variables for the all respondents and compares food system workers and non-food system workers. Of note, we find that food system workers were significantly more likely to have incomes of below $50,000 at the time of survey completion (*p* = 0.016). We also find that food system workers were significantly more likely than non-food system workers to have participated in the Supplemental Nutrition Assistance Program (SNAP) in the last 30 days (*p* = 0.002), although rates of food insecurity between groups for the same time period were not significantly different. However, we find significantly higher rates of food insecurity among food system workers than non-food system workers (34.2% vs. 19.5%, *p* = 0.033) when we examine the full period since June 2020. Additionally, we observe significantly higher rates of perceived stress among food system workers (*p* = 0.030) as compared to non-food system workers. Results of individual food security and perceived stress scale items are presented in Supplementary Table 2.

**Table 2:** Characteristics of self-identified food system workers compared to non-food system workers in Vermont.

Variable	Full sample (*N* = 441)	Food system workers (*n* = 41), n (%)	Non-food system workers (*n* = 400), n (%)	*p* value
Income at time of survey				0.016
*Under 50 k*	175 (42.1)	24 (60.0)	151 (40.2)	
*Over 50 k*	241 (57.9)	16 (40.0)	225 (59.8)	
Job disruptions				0.092
*Any job change*	170 (39.0)	21 (51.2)	149 (37.7)	
*No job change*	266 (61.0)	20 (48.8)	246 (62.3)	
Food security last 30				0.244
*Food insecure*	78 (18.2)	10 (25.0)	68 (17.5)	
*Food secure*	350 (81.8)	30 (75.0)	320 (82.5)	
Food security since June 2020				0.033
*Food insecure*	87 (20.8)	13 (34.2)	74 (19.5)	
*Food secure*	331 (79.2)	25 (65.8)	306 (80.5)	
PSS score				0.030
*Average (st. dev)*	5.6837 (3.18)	6.7073 (3.08)	5.5758 (3.17)	

For the following variables, missing values in the full sample result in smaller samples: income (*n* = 416), food security last 30 (*n* = 428), food security since June 2020 (*n* = 418), PSS score (*n* = 430).

### 3.1 Food insecurity and perceived stress of food system workers

We also use regression models, both unadjusted and adjusted, to examine the rates of food insecurity and perceived stress among food system workers. When we predict food insecurity in the past 30 days based on food system work, we find no significant relationship in either unadjusted (OR = 1.569, *p* = 0.247) or adjusted models (OR = 0.971, *p* = 0.947). In our unadjusted model predicting food insecurity since June 2020, we find a significant association with food system work (OR = 2.150, *p* = 0.036), but this association disappears when we adjust for income and job disruptions (OR = 1.633, *p* = 0.239). By linear regression, we find a significant association between food system work and perceived stress in unadjusted models (*β* = 1.131, *p* = 0.029), but the significance does not hold in our adjusted model (*β* = 0.811, *p* = 0.114; Table 3).

**Table 3:** Logistic regression models predicting food insecurity in the last 30 days and since June 2020.

Variable	Unadjusted analysis					Adjusted analysis				
	b	SE	CI	OR	*p*	b	SE	CI	OR	*p*
Food insecurity last 30										
Food system workers (ref = nonfood system workers)	0.450	0.3888	−0.312–1.212	1.569	0.247	−0.029	0.4346	−0.881–0.823	0.971	0.947
Food insecurity since June 2020										
Food system workers (ref = nonfood system workers)	0.766	0.3657	0.049–1.482	2.150	0.036	0.490	0.4162	−0.325–1.306	1.633	0.239

Unadjusted and adjusted logistic regression models are presented above. Adjusted models include income and job loss or reduction.

### 3.2 Qualitative comments by food system workers

When asked to reflect on the impact of their work on their sense of well-being during the COVID-19 pandemic, 51% (*n* = 21) of food system workers agreed that they felt their work had compromised their well-being at some point since the beginning of the pandemic. Of these participants, 20 offered further comments on the subject. All but two responses included comments on elevated COVID-19 exposure risk related to their work. Just under half of these reflected worries that work conditions necessitated increased “contact with non-household people.” One respondent summarized that shared fear simply, stating that “our potential exposure to other people was much higher.” The remainder of respondents referenced more specific experiences of elevated exposure risk at work. Among these were perceived failures by management to implement adequate protections. One respondent noted, “there have been multiple positive cases at my work as well, and I have been required to work after a coworker tested positive.” Another reported that “I felt very unsafe because of how some customers were acting and our management did not support safety measures.” Specific behaviors both by members of the public and by coworkers also troubled respondents. “People were coming in sick,” said one respondent, while another noted that “not all employees at [fast food employer] wore masks properly. We had several complaints from health inspectors.” Another individual remembered “people coming in not wearing masks and having to tell them as they yell and threaten you.”

However, in some cases, employment in the food system provided unique positive opportunities for workers. A little over a third of food system workers reported that their work had offered additional pathways to access food during the COVID-19 pandemic. Of these, 14 provided examples which typically involved financial bonuses including incentives such as employee discounts and free gift cards. Other respondents received food directly. For instance, one reported being “able to obtain foods we grew at the farm,” while others reported taking home food in a retail setting, typically when it could no longer be sold. In some cases, respondents could access multiple benefits: “I am allowed a meal every day and take home food when it is past selling date.” Shift meals and meals at sponsored work events were available to several participants. Outside of these direct pathways, one respondent referenced the value of knowledge obtained through their work role, noting that she “always [knew] when the next food event will be.” Still, 63% (*n* = 26) of participants did not report receiving any additional food access benefits through their work in the food system (Table 4).

**Table 4:** Generalized linear regression model predicting perceived stress in the last 30 days since survey completion.

Variable	Unadjusted analysis				Adjusted analysis			
	b	SE	CI	*p*	b	SE	CI	*p*
PSS score								
Food system workers (ref = nonfood system workers)	1.131	0.5182	0.116–2.147	0.029	0.811	0.5126	−0.194–1.816	0.114

Unadjusted and adjusted linear regression models are presented above. Adjusted models include income and job loss or reduction.

Food system workers were additionally given the opportunity to provide further comments on their work life during the COVID-19 pandemic. A few reflected on the value of their work during this time. For example, one stated that “obviously [it] was not the best time to be a restaurant owner, but it was good to be able to help some people with their meals.” Likewise, another felt that “we are helping the community feed their families without having to come into the store.” Others expressed disillusionment or frustration with their work: “It was awful” stated one respondent. “Customers were hostile to me for wearing a mask and I felt at risk.” Another was even more succinct, merely expressing: “People suck.” For one food system worker, their experiences were negative enough to change their entire outlook on the field: “previously to the COVID-19 pandemic working in food service was my job of choice, but I am currently trying to get out of the industry and NEVER want to return to working with food.”

### 3.3 Perceptions of food system workers

**Figure 1:**
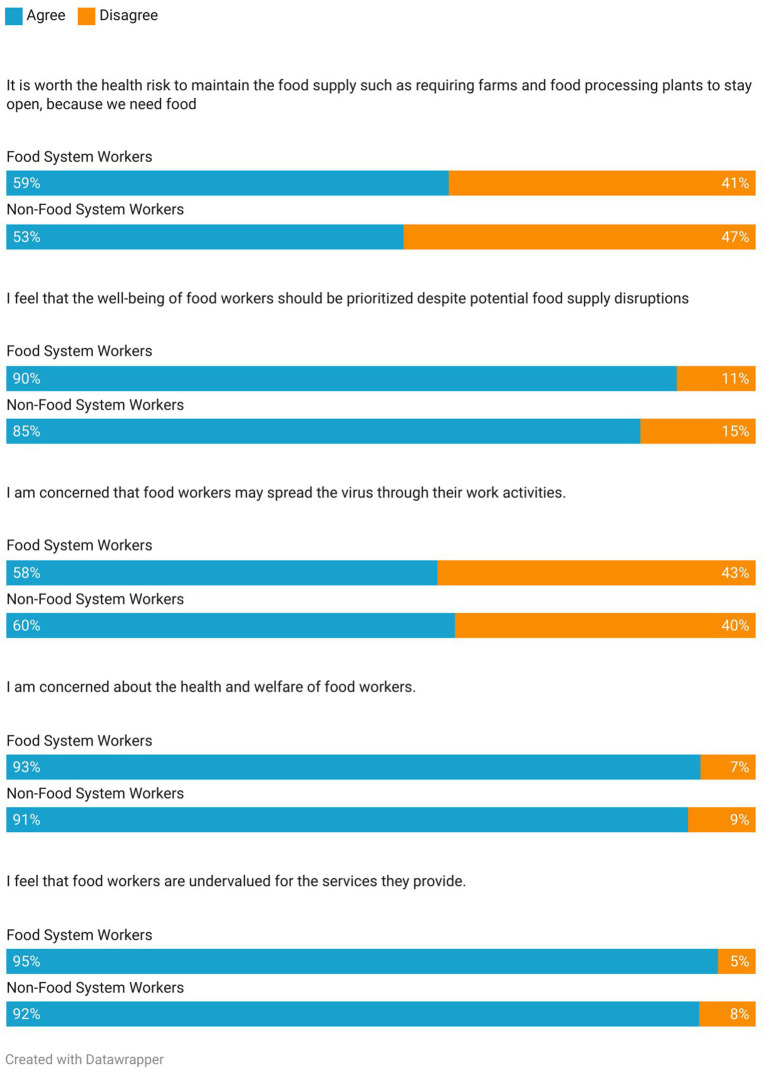
Perspectives on food system workers by employment type. For the following variables, missing values in the full sample result in smaller samples: food supply concerns (*n* = 390), worker well-being (*n* = 408), workers spread virus (*n* = 408), workers health (*n* = 422), workers undervalued (*n* = 422).

We additionally asked participants several questions about how they felt about food system workers and their roles during the pandemic. We found no statistically significant differences in the responses from FS workers and non-FS workers to these questions, although in some cases there were too few responses from FS workers to perform statistical analyses. Most respondents agreed that they were concerned about the health and welfare of food system workers, that food system workers were undervalued, and that the well-being of food system workers should be prioritized, despite potential food supply disruptions (Figure 1). However, when asked whether “it is worth the health risk to maintain the food supply such as requiring farms and food processing plants to stay open, because we need food,” more than half of respondents, including food system workers, agreed. Additionally, more than half of respondents in both categories agreed that they were concerned that food system workers may spread the virus through their work activities.

Eighty participants (74 non-food system workers and 6 food system workers) provided additional comments on food system workers. More than half of all comments deal with health and safety concerns for food system workers, especially in the context of the COVID-19 pandemic (i.e., “after healthcare workers, I believe food system workers should have been prioritized for the vaccine given how critical their work was and is to keeping society functioning”) but occasionally in a more general capacity (i.e., “I think that factory farming and processing facilities owners need to be regulated in a manner which protects the wellbeing of workers and consumers as intended, not just profits and efficiency as regulation enforcement has become.”). A second key theme within the comments related to the need for financial supports for those working in the food system. Those that focused on this topic likewise spanned calls for targeted support in response to the pandemic, such as “I’m upset that the hero pay was limited to a few months, when the pandemic and risks are still ongoing,” in addition to more broad comments on the compensation of food system workers: “The $15 minimum wage would greatly help in providing security to food system workers from the fields to the stores.” Another observed that “food system workers were given a lot of lip service on how they were essential workers, but not generally given benefits such as more pay.” Other comments (10% or less) focused on rights for migrant workers and broad structural observations on the food system. Seven participants discussed the challenges of balancing the dual aims of protecting workers and maintaining the food supply, which were perceived to be in tension: “It’s a balancing act, as we need to eat. I think that more safeguards need to be taken to protect workers on the job.” Only three respondents expressed worry over how the actions of food system workers during the pandemic might affect them.

## 4 Discussion

Food system workers are vital to a functioning food system and, as such, are integral in ensuring the food security of the general public, but a healthy food system requires healthy workers in order to function optimally. Nonetheless, data suggest that food system workers experience food insecurity, as well as some indicators of poor mental health, at higher rates than the general public (11, 15, 32, 34). This study expands on the literature by exploring the food security and well-being of food system workers in Vermont at a unique period in history, during which the integrity of the food system was at risk and rates of food insecurity rose overall, rendering these workers even more critical. Food system workers often found themselves at the front line of the pandemic without adequate training, equipment, and policies in place to protect their health, and with limited compensation for the services they provide (3). We find higher rates of perceived stress and food insecurity among food system workers when we examine the full time period since June 2020. However, these relationships are not maintained when we control for income and multiple forms of job loss and reduction, suggesting that the associations were primarily due to high rates of economic vulnerability among food system workers. This aligns with prior literature finding overall lower incomes among food system workers (20). Compared to workers in other industries deemed “essential” during the pandemic (e.g., healthcare, emergency services), food system workers have lower incomes, are less likely to be unionized, and come disproportionately from socio-economically disadvantaged groups, underscoring the importance of understanding their experiences (42).

Interestingly, although in our adjusted regression models there is no significant association between food system work and food insecurity for any period, even in unadjusted models this trend is significant only for the full period since June 2020, and not in the 30 days preceding survey completion. Given the influential role of economic factors suggested by our data, the discrepancy in significance between unadjusted models may be reflective of the overall economic environment experienced by workers during these periods. Whereas the full period since June 2020 encompasses an intermediate phase of the pandemic wherein the economic ripple effects of shutdowns and early layoffs were highly prevalent, the 30 days prior to survey completion represent a period of greater economic stability, marked by fewer restrictions and many households having received stimulus payments (43) and other pandemic-related benefits (44). These changes may correspond to greater job security for food system workers, which may have translated into lower rates of food insecurity. This theory seems particularly plausible when we consider fluctuations in the restaurant industry over the course of this time. Likewise, prior literature has found that employment reductions were common in the food service industry during the early pandemic (29), but this trend may have become less prominent later in the pandemic. Notably, although overall trends suggest higher rates of food insecurity among food system workers, our qualitative data reveal that, in some cases, food system work can offer additional pathways to accessing food. However, only about a third of our respondents reported benefiting from such pathways, and they are likely not enough to overcome economic disadvantages experienced by food system workers in general.

Based on our data, it is reasonable to suggest that higher rates of perceived stress among food system workers are also, at least in part, related to economic concerns. However, the impact of stressors associated with elevated exposure risks taken on by frontline food system workers should not be ignored, as is evident in our qualitative results. Workers in the food service industries, particularly tipped workers, may be subject to the whims of the customer and client, even when it puts them at risk. This is always true, but the potential detrimental impacts are particularly pronounced under pandemic conditions, with serious corporeal risks compounded by limited feelings of agency. Nearly half of food system workers who participated in our survey reported feeling that their well-being was compromised in this way. Of note, our analyses grouped food system workers from diverse fields to maximize our sample size; were we to focus exclusively on recruiting front line food system workers, we may find these patterns even more prevalent.

In light of these results, the widespread support for the rights and welfare of food system workers expressed by our participants is notable. Are these concerns for the well-being of food system workers a novel response to vulnerabilities highlighted by the COVID-19 pandemic? And what barriers might prevent these feelings of support from translating into more direct action? As one survey respondent noted, “food system workers were given a lot of lip service on how they were essential workers, but not generally given benefits such as more pay.” While the sentiment of support for the welfare of food system workers is clearly present, survey respondents also recognized the pivotal role of these workers in ensuring health and safety for all. Notably, when we asked participants if they felt that the well-being of food workers should be prioritized despite potential food supply disruptions, 85% of respondents agreed. Yet over half of the sample (53.6%) agreed that it is worth the health risk to require farms and food processors to stay open to maintain the food supply. Several participants directly grappled with these conflicting values in open-ended comments, acknowledging the difficulty of preserving the health and well-being of workers without potentially putting the stability of the entire food supply at risk. Put very simply by one participant, “it’s a balancing act, as we need to eat.” This apparent dissonance offers an opportunity to reflect and reexamine how our food system might be better structured to simultaneously support both of these aims while these issues are at the forefront of our collective consciousness.

As our sample is drawn from a survey that explores the experiences of Vermonters in diverse fields, the relatively small subsample of food system workers precludes extensive analyses of demographic characteristics such as race and ethnicity or comparison of our outcomes across different types of food system workers. Additionally, it should be noted that our sample is not representative and certain groups may be overrepresented (i.e., female gender). Grouping food system workers in the manner described above allows us to comment more broadly on the experiences of diverse types of food workers but limits our ability to comment on any specific category of worker. Further, we did not ask about forms of essential work and comparison of the experiences of food system workers vis-à-vis other types of essential workers is warranted.

Understanding factors that impact the well-being of food system workers is essential if we hope to optimize the resilience of our food system under precarious conditions. When economic and social conditions drive some workers out of the industry and compromise the safety and security of those that remain, a reexamination of the institutions that support or fail to support these workers is called for to ensure that continuity and strength of the systems that feed us all.

## Data availability statement

The de-identified data supporting the conclusions of this article will be made available by the authors to researchers who complete appropriate human ethics training, without undue reservation.

## Ethics statement

The studies involving humans were approved by University of Vermont Committee on Human Research in the Behavioral and Social Sciences. The studies were conducted in accordance with the local legislation and institutional requirements. The ethics committee/institutional review board waived the requirement of written informed consent for participation from the participants or the participants’ legal guardians/next of kin because the survey participants provided inferred consent online by continuing to take the survey after the information sheet/introduction screen and affirming they were over the age of 18.

## Author contributions

ES: Conceptualization, Formal analysis, Writing - original draft. MN: Conceptualization, Funding Acquisition, Writing - review & editing. FB - Conceptualization, Funding Acquisition, Writing - review & editing. TM: Writing - review & editing. EB: Conceptualization, Funding Acquisition, Writing - review & editing.
