# Supplementary material for: Higher rates of food insecurity and stress experienced by food systems workers during the first year of the COVID-19 pandemic
Source: Front Nutr. 2024 May 7;11:1274656. doi: 10.3389/fnut.2024.1274656 (PMC11106489; doi:10.3389/fnut.2024.1274656)
Supplement: Supplementary file 1 [file Table_1.DOCX]

Supplementary Material

**Higher Rates of Food Insecurity and Stress Experienced by Food Systems Workers during the First Year of the COVID-19 Pandemic**

**Emma H. Spence, Meredith T. Niles, Farryl Bertmann, Teresa Mares, Emily H. Belarmino***

**Correspondence:** Emily H. Belarmino: [Emily.belarmino@uvm.edu](mailto:Emily.belarmino@uvm.edu)

**Supplementary Table 1. Characteristics of self-identified food system workers in Vermont by employment type.**

| **Variable** | **All food system workers (n=41), n (%)** | **Food service (n=16)** | **Food retail (n=10)** | **Food processing (n=8)** | **Agriculture (n =6)** | **Other (n=3)** |
| --- | --- | --- | --- | --- | --- | --- |
| Income at time of Survey  *Under 50k*  *Over 50k* | 24 (60.0)  16 (40.0) | 9 (56.3)  7 (43.8) | 6 (60.0)  4 (40.0) | 6 (75.0)  2 (25.0) | 2 (33.3)  4 (66.6) | 2 (100.0)  0 (0.0) |
| Job Disruptions  *Any job change*  *No job change* | 21 (51.2)  20 (48.8) | 10 (62.5)  6 (37.5) | 4 (40.0)  6 (60.0) | 7 (87.5)  1 (12.5) | 0 (0.0)  6 (100.0) | 1 (33.3)  2 (66.6) |
| Food Security Last 30  *Food Insecure*  *Food Secure* | 10 (25.0)  30 (75.0) | 4 (25.0)  12 (75.0) | 2 (22.2)  7 (77.8) | 3 (37.5)  5 (62.5) | 0 (0.0)  6 (100.0) | 0 (0.0)  3 (100.0) |
| Food Security since June 2020  *Food Insecure*  *Food Secure* | 13 (34.2)  25 (65.8) | 4 (28.6)  10 (71.4) | 3 (30.0)  7 (70.0) | 5 (62.5)  3 (37.5) | 1 (16.7)  5 (83.3) | 0 (0.0)  2 (100.0) |
| PSS Score  *Average (st. dev)* | 6.7 (3.1) | 7.0 (3.4) | 7.2 (3.4) | 7.4 (1.7) | 5.8 (3.2) | 7.3 (2.9) |

Note. For the following variables, missing values result in smaller samples in each category: income (n=40), food security last 30 (n=40), and food security since June 2020 (n=38).

**Supplementary Table 2. Responses to individual food security and perceived stress scale items.**

| **Variable** | **Full Sample (N=441), *M (SD)*** | **Food System Workers (n=41), *M (SD)*** | **Non-Food System Workers (n=400), *M (SD)*** |
| --- | --- | --- | --- |
| Perceived Stress Score |  |  |  |
| 1. In the last month, how often have you felt that you were unable to control the important things in your life? (0=Never, 1=Almost never, 2=Sometimes, 3=Fairly often, 4=Very often) | 1.73 (1.07) | 1.93 (0.848) | 1.70 (1.09) |
| 1. In the last month, how often have you felt confident about your ability to handle your personal problems? (0=Very often, 1=Fairly often, 2=Sometimes, 3=Almost never, 4=Never) | 2.88 (1.00) | 2.54 (1.05) | 2.91 (0.99) |
| 1. In the last month, how often have you felt that things were going your way? (0=Very often, 1=Fairly often, 2=Sometimes, 3=Almost never, 4=Never) | 2.49 (0.86) | 2.37 (0.77) | 2.50 (0.87) |
| 1. In the last month, how often have you felt difficulties were piling up so high that you could not overcome them?  (0=Never, 1=Almost never, 2=Sometimes, 3=Fairly often, 4=Very often) | 1.32 (1.06) | 1.68 (1.04) | 1.28 (1.06) |
| Food Security Since June |  |  |  |
| 1. The food that my household bought just didn't last, and I/we didn't have money to get more. (0=No, 1=Yes) | 1.19 (0.45) | 1.37 (0,58) | 1.18 (0.43) |
| 1. I/we couldn't afford to eat balanced meals. (0=No, 1=Yes) | 1.23 (0.49) | 1.41 (0.59) | 1.2 (0.48) |
| 1. Did you or other adults in your household ever cut the size of your meals or skip meals because there wasn't enough money for food? (0=No, 1=Yes) | 0.15 (0.36) | 0.29 (0.46) | 0.14 (0.35) |
| 1. Did you ever eat less than you felt you should because there wasn't enough money for food? (0=No, 1=Yes) | 0.15 (0.36) | 0.24 (0.44) | 0.14 (0.35) |
| 1. Were you ever hungry but didn't eat because there wasn't enough money for food? (0=No, 1=Yes) | 0.10 (0.30) | 0.24 (0.44) | 0.09 (0.28) |
| 1. How often did you cut the size of your meals or skip meals? (1=Only 1 or 2 months, 2=Some months but not every month, 3=Almost every month) | 2.25 (0.67) | 2.50 (0.52) | 2.20 (0.69) |
| Food Security in the Last 30 Days |  |  |  |
| 1. The food that my household bought just didn't last, and I/we didn't have money to get more. (0=No, 1=Yes) | 1.13 (0.39) | 1.18 (0.39) | 1.12 (0.39) |
| 1. I/we couldn't afford to eat balanced meals. (0=No, 1=Yes) | 1.18 (0.45) | 1.25 (0.44) | 1.17 (0.45) |
| 1. Did you or other adults in your household ever cut the size of your meals or skip meals because there wasn't enough money for food? (0=No, 1=Yes) | 0.09 (0.283) | 0.15 (0.36) | 0.08 (0.27) |
| 1. Did you ever eat less than you felt you should because there wasn't enough money for food? (0=No, 1=Yes) | 0.09 (0.29) | 0.15 (0.36) | 0.09 (0.28) |
| 1. Were you ever hungry but didn't eat because there wasn't enough money for food? (0=No, 1=Yes) | 0.07 (0.26) | 0.10 (0.30) | 0.07 (0.25) |
| 1. How often did you cut the size of your meals or skip meals? (Number of days) | 12.31 (9.91) | 9.33 (4.76) | 12.90 (10.61) |

Note. For the following variables, missing values result in smaller samples in each category: all perceived stress items (n=430 for full sample, n=389 for non-food system workers); food security since June (Q1: n=437 for full sample, n=396 for non-food system workers; Q2: n=433 for full sample, n=392 for non-food system workers; Q3: n=433 for full sample, n=392 for non-food system workers; Q4: n=431 for full sample, n=390 for non-food system workers; Q5: n=433 for full sample, n=392 for non-food system workers; Q6: n=36 for full sample, n=12 for food system workers, n=51 for non-food system workers); food security in the last 30 days (Q1: n=425 for full sample, n=40 for food system workers, n=385 for non-food system workers; Q2: n=421 for full sample, n=40 for food system workers, n=381 for non-food system workers; Q3: n=433 for full sample, n=392 for non-food system workers; Q4: n=428 for full sample, n=387 for non-food system workers; Q5: n=432 for full sample, n=391 for non-food system workers; Q6: n=36 for full sample, n=6 for food system workers, n=30 for non-food system workers)
